# Norovirus Outbreaks from Drinking Water

**DOI:** 10.3201/eid1111.050487

**Published:** 2005-11

**Authors:** Leena Maunula, Ilkka T. Miettinen, Carl-Henrik von Bonsdorff

**Affiliations:** * HUCH Laboratory Diagnostics, Helsinki, Finland; † University of Helsinki, Helsinki, Finland; ‡ National Public Health Institute, Kuopio, Finland

**Keywords:** Norovirus, gastroenteritis, reverse transcriptase polymerase chain reaction, genotype, disease outbreaks, research

## Abstract

As part of an intensified monitoring program for foodborne disease outbreaks in Finland, waterborne outbreaks were investigated for viruses. The diagnostic procedure included analysis of patients' stool samples by electron microscopy and reverse transcription–polymerase chain reaction (RT-PCR) for noroviruses and astroviruses. When these test results were positive for a virus, the water sample was analyzed. Virus concentration was based on positively charged filters from 1-L samples. Of the total 41 waterborne outbreaks reported during the observation period (1998–2003), samples from 28 outbreaks were available for analysis. As judged by RT-PCR results from patient samples, noroviruses caused 18 outbreaks. In 10 outbreaks, the water sample also yielded a norovirus. In all but 1 instance, the amplicon sequence was identical to that recovered from the patients. The ubiquity of waterborne norovirus outbreaks calls for measures to monitor water for viruses.

## Abstract 2

Norovirus contamination calls for viral monitoring of drinking water.

Water can be a source of disease outbreaks (*1*). Contamination takes place almost exclusively by sewage that contains enteric pathogens, and enteric viruses that affect humans are mostly species-specific; their abundance may be explained by high concentrations in the stool of patients. Noroviruses (previously called Norwalk-like viruses) cause gastroenteritis in all age groups. Since noroviruses, unlike enteroviruses, do not easily grow in cell culture, their role became evident only in the 1990s, when specific diagnostic methods became available. Only in recent years has the vast genomic variety of noroviruses become apparent (*2*). A recent report (*3*) lists 5 genogroups and 22 genetic clusters that include mostly human but also porcine and murine viruses.

In addition to numerous community-based outbreaks, in which transmission is thought to take place from person to person, outbreaks caused by contaminated food have been frequent (*4*). The dominant role of noroviruses in foodborne and waterborne outbreaks has been estimated by Mead et al. (*5*). Several waterborne outbreaks have been detected on the basis of epidemiologic evidence (*6**,**7*), and only in 1997 did the first report of noroviruses in well water appear (*8*). The genome-based diagnostic procedure, i.e., reverse transcriptase–polymerase chain reaction (RT-PCR), offers a sensitive and specific tool to identify these viruses. Sequence-based identification is effective for source-tracking outbreaks, especially those caused by noroviruses, which show a highly variable nucleotide sequence even within the short amplicon produced in the polymerase region of the virus (*9*).

Waterborne viral outbreaks are often difficult to recognize. Illness caused by norovirus is common, and if the contamination level is low, the number of cases remains low. A rather extensive outbreak is usually required for medical personnel and authorities to recognize water as a possible source of infection (*10*). This report includes virologic analyses of Finnish waterborne outbreaks during a 6-year period. We describe an improved procedure to identify water as the source of viral outbreaks.

## Methods

Reporting of foodborne and waterborne outbreaks in Finland was reorganized and intensified in 1997; new regulations emphasized that all suspected cases should be immediately reported to the National Public Health Institute (KTL). Recommendations were given for properly collecting both patient and environmental samples. The functions of local outbreak investigation teams were clarified and included training in conducting epidemiologic surveys. Laboratory performance was improved by including options for viral and protozoan diagnostics from both patient and environmental samples. All cases in which water was suspected as the source of the outbreak were reported to KTL. Sampling recommendations included 3–10 representative patient stool samples. Water samples, raw water, and when appropriate, tap water from different parts of the distribution network were collected immediately. Despite recommendations, not all outbreaks were investigated for viruses. The criteria for establishing an outbreak as waterborne were according to the English classification (grades A–D) (*11*).

In total, 271 patient samples from 25 outbreaks were analyzed for viruses. The range of fecal samples obtained from each outbreak was 2–69 (mean 11). A 10% fecal suspension in 0.05 mol/L Tris-HCl, 0.1 mol/L NaCl, 1 mmol/L CaCl_2_, pH 7.4, was used for RNA extraction.

A total of 73 water samples from 27 outbreaks were analyzed; 1- to 2-L water samples, collected in clean glass or plastic bottles, were concentrated as described by Gilgen et al. (*12*). The 1-L samples were run through a positively charged disk membrane filter (diameter 47 mm, pore size 45 μm; AMF-Cuno, Zetapor, Meriden, CO, USA) with or without a fiberglass prefilter. After the elution step in 50 mmol/L glycine buffer, pH 9.5, containing 1% beef extract, the eluate was rapidly neutralized with HCl. The volume was further reduced to ≈100 μL with a microconcentrator (Centricon-100, Amicon, Beverly, MA, USA). This sample was used for RNA extraction and PCR as described (*10*).

RNA extraction and RT-PCR for the norovirus polymerase region were performed as described (*13*). Briefly, RNA was extracted by using phenol- and guanidine thiocyanate–containing Tripure reagent (Roche, Indianapolis, IN, USA) and precipitated with ethanol. Viral RNA was transcribed to cDNA, and DNA amplification was performed in separate tubes for norovirus genogroups I and II (GI and GII) by manual PCR with primers Nvp110 (*14*) and N69 (*15*), and Nvp110 and NI (*16*), respectively. From 2002 on, the forward primers for the genogroups were modified as KA1 (5´-GANGGCCTSCCMTCWGGNTT-3´) and KA2 (5´-TGGAATTCNATHGCCCAYTGG-3´). The amplicons were visualized by electrophoresis in an agarose gel, hybridized by a probe panel, and used for nucleotide sequencing.

Sequencing was performed manually (Sequenase, version 2.0 DNA sequencing kit, USB, Cleveland, OH, USA) as described (*13*). Sequence analysis was performed by programs SeqApp and ClustalW. Our sequences were aligned with the following EMBL/GenBank noroviruses: Southampton/91/UK (L07418), Norwalk/68/US (M87661), Malta (AJ277616), Melksham/94/UK (X81879), Hawaii/76/US (U07611), Lordsdale/93/UK (X86557), GIIb (AY7732101), GIId (AF312728), and murine norovirus (AY228235). For nucleotide sequences for Hillingdon/94/UK and Grimsby/95/UK, see Vinje et al (*17*); sequence of Lord Harris comes from the sequence database of the European network (*9**,**18*). GenBank accession numbers for nucleotide sequences of this study are AY958213–9 for GI and AY958204–12 for GII noroviruses.

## Results

### Description of Outbreaks and Viral Findings

In total, 41 waterborne outbreaks (3–11 per year) were registered in Finland from 1998 to 2003. Of these, 28 (61%) were investigated for viruses. In 24 outbreaks both water and patient samples were available for analysis; in 3 outbreaks only water was available, and in 1 outbreak only patient samples were available for analysis. Samples for viral analysis were not obtained from the remaining 13 outbreaks. Analysis was performed by RT-PCR. Patient samples were also screened by electron microscopy for other enteric viruses and analyzed by RT-PCR for astroviruses. For water samples, a concentration method according to Gilgen et al. (*12*) was established, starting from the volume of 1 L. In most cases, water samples were analyzed only for noroviruses. The most prominent viruses that caused waterborne outbreaks were noroviruses (18 outbreaks). Rotavirus caused 1 waterborne outbreak, and no viruses were found in 9 epidemics. Bacterial findings will be published elsewhere.

The 18 waterborne norovirus outbreaks are summarized in Table 1. In every year except 2001, several norovirus outbreaks occurred in Finland. During the study period, 6 large norovirus epidemics with >200 cases were encountered. In the largest epidemics, >10,000 persons were exposed, and 2,000–5,500 cases occurred; in addition, 7 medium-sized (40–100 cases) and 5 small outbreaks (<20 cases) were caused by noroviruses.

**Table 1:** Suspected and identified norovirus outbreaks, Finland, 1998–2003

Outbreak	Date	No. exposed/no. ill	Water source
E1, community*	Mar 1998	5,000/2,500	Surface water used as tap water
E2, community	Apr 1998	15,000/2,000	Ground water
E3, rental camp cottage	Jul 1998	45/13	Well
E4, camp on island	Aug 1998	120/40	Communal, well
E5, community	Jan 1999	2,500/200	Ground water
E6, factory area	Feb 1999	250/100	Ground water
E7, community	Apr 1999	160/58	Ground water
E8, spa	Jul 1999	100/60	Well
E9, community*	Mar 2000	10,000/5,500	Ground water
E10, private household	Aug 2000	14/13	Well (drilled)
E11, community	Dec 2000	2,200/300	Ground water
E12, farm (for guests)	Apr 2002	50/25	Well (dug)
E13, community	Oct 2002	960/300	Ground water
E14, guest house	Feb 2003	13/11	Lake water used for drinking
E15, community	May 2003	150/95	Well (drilled)
E16, rental cottage	Aug 2003	25/20	Well
E17, holiday camp	May 2003	56/40	Surface water (river)
E18, community	Apr 2003	90/40	Ground water, broken pipe

*Detailed descriptions of the epidemics E1 and E9 have been published (*10**,**19*).

Most norovirus contaminations occurred in groundwater systems, which are used most commonly in Finland. In 3 instances, surface, lake, or river water was used. Of the ground water epidemics, 8 occurred in public communal systems and 7 in private ground water wells. Typically rental cottages or different kinds of camping grounds with their own wells were affected.

**Figure 1:**
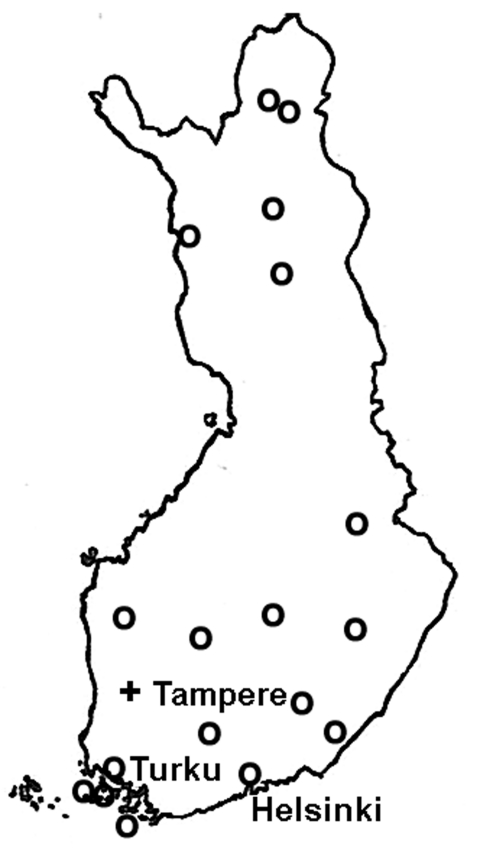
Map of Finland; circles indicate distribution of waterborne norovirus outbreaks, 1998–2003.

**Figure 2:**
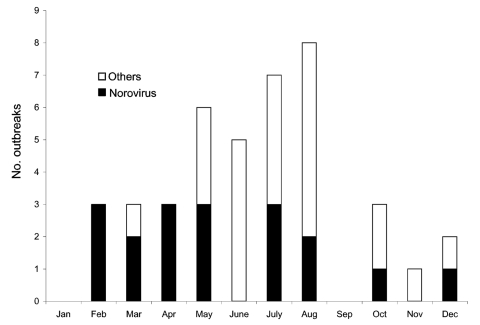
Monthly distribution of waterborne outbreaks, including norovirus outbreaks, Finland, 1998–2003.

The geographic distribution of the waterborne norovirus outbreaks is shown in Figure 1. Outbreaks occurred all over the country, from the southern archipelago to the northernmost parts of Finland. Seasonal risk for waterborne norovirus outbreak seemed to be approximately equal (Figure 2). Half (20 of 41) of the waterborne epidemics occurred in summer, and norovirus outbreaks (11 of 15) were most common in late winter to spring (February–May). In fact most outbreaks in winter were caused by noroviruses, while in summer they were mainly caused by bacteria.

### Detailed Analysis of Noroviruses

Noroviruses from 16 outbreaks (E1–E16) were further characterized by sequence analysis of amplicons, from which the genotype was also deduced (Table 2). Noroviruses appeared in the patient samples in all 16 outbreaks and in water samples of 10 epidemics. Coliforms were also present in 9 epidemics, whereas in 7 outbreaks, no indication of microbiologic contamination was seen. Most outbreaks were caused by a single norovirus strain/genotype (11 epidemics); >1 virus was found more often in large outbreaks than in small ones. GII noroviruses were only slightly more common than GI (7 vs. 5 outbreaks). Of the 10 epidemics with positive water samples, equal numbers of GI and GII genotypes were detected.

**Table 2:** Findings in suspected and identified norovirus outbreaks, Finland, 1998–2003

Outbreak	Date	Presence of coliforms	Microbiologic findings (genotype)*	
			Patients	Water
E1, community	Mar 1998	–	GI.6, GII.4	GII.4
E2, community	Apr 1998	–	GI.6, GIId	–
E3, rental camp cottage	Jul 1998	–	GI, GII	–
E4, camp on island	Aug 1998	–	GII.5	–
E5, community	Jan 1999	–	GII.4	–
E6, factory area	Feb 1999	+	GI.3	GI.3
E7, community	Apr 1999	+	GI.6	GI.6
E8, spa	Jul 1999	+	GI.3	GI.3
E9, community	Mar 2000	–	GI.3, GII	–
E10, private household	Aug 2000	+	GI.3	GI.3
E11, community	Dec 2000	+	GII.4	GII.4, GII.1
E12, farm (for guests)	Apr 2002	+	GII.NA	GII.NA
E13, community	Oct 2002	+	GIIb	–
E14, guest house	Feb 2003	–	GII.4nv	GII.4nv
E15, community	May 2003	+	GII.4nv	GII.4nv
E16, rental cottage	Aug 2003	+	GI.6	GI.6

*nv, new variant.

**Figure 3:**
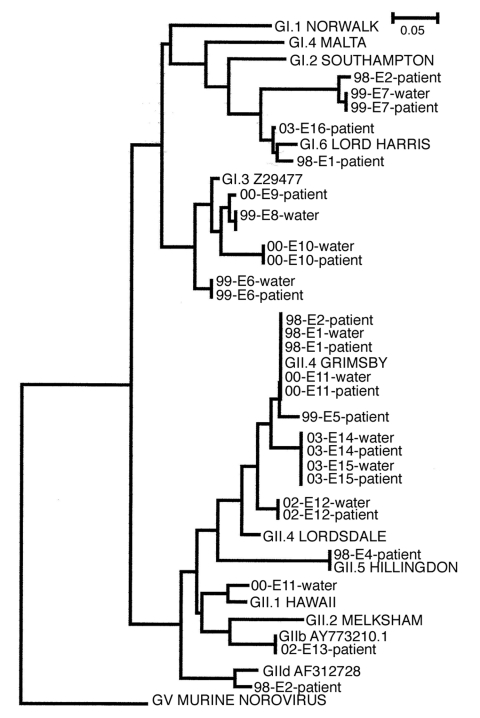
Phylogenetic trees derived from 28 norovirus nucleotide sequences from the polymerase region. The nucleotide sequences were from 10 water and 18 patient samples of 14 outbreaks. Trees were constructed by using the neighbor-joining method with the ClustalW software package. Scale indicated by bars. Branch lengths are related to degree of divergence between sequences.

In all but 1 of these outbreaks, the same norovirus genotype found in water samples also appeared in patient samples. The only exception was epidemic E11, in which 2 norovirus sequences, GII.1 and GII.4, were detected in the water sample, but only type GII.4 was detected in the patient samples. Not only the viral genotype but also the entire amplicon sequence were identical in each outbreak (Figure 3). Two norovirus genogroup I types, GI.3 (Birmingham) and GI.6 (Sindlesham, Hesse), were found; 1 GI sequence (outbreak E3) remained undetermined.

In the GII outbreaks, at least 4 different genotypes were found in patient or water samples. The most common genotype was GII.4 (Bristol, Lordsdale), found in water samples of 4 epidemics, and beginning in 2003, it was the new variant type (*20*). The established genotypes GII.1 (Hawaii) and GII.5 (Hillingdon) were also detected in some outbreaks, along with some potentially new genotypes or sequences that did not cluster well in any of the established genotypes, such as GIId (Upinniemi) and GIIb. As Figure 3 shows, in most outbreaks a virus with a unique amplicon sequence was recovered, even when it belonged to the same genotype as viruses in the other waterborne outbreaks. Norovirus genotype GII.4 was the only exception, and a longer nucleotide sequence likely would have shown some genetic differences (detected between sequences of epidemics E1 and E11; data not shown).

## Discussion

As part of the improved and intensified outbreak surveillance system in Finland, we have identified waterborne viral outbreaks since 1998. In a relatively brief period, during which norovirus diagnostics have been available for patient as well as environmental samples, a considerable number of waterborne norovirus outbreaks have been detected.

That Finland has >1,300 water treatment plants may in part explain the numerous outbreaks. Many of these plants still use surface water (lakes or rivers) as raw water. Inadequate disinfection is then the most common reason for waterborne epidemics, as was the case in outbreak E1 (*10*). At risk also are water plants that use groundwater and no disinfection. In Finland, snow melts in spring while the ground is still frozen, which leads to surface runoffs and flooding. Breaks in sewer lines in the vicinity of a well caused several large waterborne outbreaks. Poor sewage disposal also caused many small waterborne outbreaks in private homes or rental cottages.

The large number of genetically distinct norovirus genotypes has been advantageous in investigating waterborne epidemics. Although the short amplicon sequence does not definitively show that 2 viruses are identical, for the purpose of source tracing it seems adequate. In this study, a unique viral sequence appeared in most norovirus outbreaks, and viruses from patients and water in a particular outbreak showed identical sequences. The success in most outbreaks in identifying a norovirus with the same sequence from patients and water may be due to the fact that the outbreaks have taken place in small communities. In large waterborne outbreaks, usually >1 norovirus strain and often other viruses and microbes are causative agents.

Both norovirus genogroups occurred in waterborne epidemics. In a 5-year study (1998–2002) in Finland, GII outbreaks clearly outnumbered those caused by GI noroviruses (86.9% vs. 13.1%) (*21*). In waterborne outbreaks, however, nearly half were caused by GI viruses. Some differences may occur in stability as well as ability to spread from person to person among viruses representing different genotypes. Type GI.3, the most common GI genotype in water samples, was also the most frequent GI type in community outbreaks (*21*). Viruses of this genotype have caused waterborne outbreaks in the United States in 2001 (*22*) and in the Netherlands (*23*).

As might be expected, keeping in mind its ubiquity (*24**,**25*), the GII.4 genotype was present in several waterborne outbreaks, and in Finland it has been the most frequent genotype in all outbreaks. The GII.4 new variant emerged in Finland in June 2002, and in the following year 2 waterborne outbreaks were caused by this new variant (*20*). Another emerging genotype, GIIb, found in Finland in 2001, a year later than in southern parts of Europe, was a causative agent in a waterborne outbreak in 2002. A waterborne outbreak in Sweden caused by this genotype has recently been reported (*26*).

Environmental virology of human pathogen detection has a rather limited history. A classic case is the monitoring of polioviruses in sewage (*27*). This method, based on a cell culture technique, is sensitive in detecting circulating wild poliovirus. Further efforts in environmental virology were lacking for many years, mainly because suitable methods were absent. Only after gene amplification techniques were introduced could a tool be developed to successfully detect norovirus in environmental samples (*8**,**10*). In recent years, an increasing number of reports have described waterborne norovirus outbreaks through contaminated drinking or recreational water (*22**,**23**,**28**,**29*).

National recommendations for volumes of water to be tested vary between tens and hundreds of liters. Such volumes pose a serious practical problem for the testing laboratory. For viral detection by RT-PCR, a smaller volume (1 L) is preferred, as suggested by Gilgen et al (*12*). Independent of the concentration method, the increase in RT-PCR inhibitors usually sets limits on the water concentration. Sensitive methods are needed to detect viruses in environmental samples. Recent reports on the applicability and sensitivity of real-time RT-PCR (30–32) for noroviruses also offer new possibilities to enhance its sensitivity. Another factor is that the test then becomes more rapid, which is essential in monitoring water quality, particularly in epidemic situations. The third advantage is that a quantitative estimate of the contamination level is obtained.

Microbial risks from water are recognized, with much emphasis on risk assessment (*33*). Assessment of water, however, depends on indicator organisms, such as coliforms or enterococci, whose survival in water is shorter than that of enteric viruses, especially norovirus and hepatitis A virus. Therefore, viruses can easily be harbored in "microbiologically immaculate" water (*34**,**35*). In situations in which a well is contaminated by sewage, coliforms are nearly always found. When sewage is released into lake water that serves as raw water downstream, indicator organisms may no longer be detectable, but noroviruses can still be present and cause illness. This sequence of events probably led to the first outbreak we examined (E1) (*10*).

When water plants use surface water, the contamination may be short-lived and may have vanished by the time the outbreak is detected. A "rolling sample" system might be used in which samples are collected in water plants at risk for contamination at regular intervals (e.g., daily, weekly) and stored at 4°C. Unless signs of an outbreak appear, the samples can be discarded at the same pace that new ones are collected. In case of contamination, water samples would be available for analyses.

The evidence presented here together with several recent reports mentioned above show the role of viruses as contaminants of drinking water. In Finland, the finding that noroviruses frequently cause waterborne outbreaks has led to authorities' increased awareness of viral risks. As a consequence, laboratory techniques have been improved, and the capacity for analyzing environmental samples, especially water, has increased. Legislative measures for viral monitoring as part of the microbial risk assessment in drinking water production should be seriously considered.
